# An early start of West Nile virus seasonal transmission: the added value of One Heath surveillance in detecting early circulation and triggering timely response in Italy, June to July 2018

**DOI:** 10.2807/1560-7917.ES.2018.23.32.1800427

**Published:** 2018-08-09

**Authors:** Flavia Riccardo, Federica Monaco, Antonino Bella, Giovanni Savini, Francesca Russo, Roberto Cagarelli, Michele Dottori, Caterina Rizzo, Giulietta Venturi, Marco Di Luca, Simonetta Pupella, Letizia Lombardini, Patrizio Pezzotti, Patrizia Parodi, Francesco Maraglino, Alessandro Nanni Costa, Giancarlo Maria Liumbruno, Giovanni Rezza, the working group

**Affiliations:** 1 Department of Infectious Diseases, National Institute of Health, Rome, Italy; 2 Istituto Zooprofilattico Sperimentale Abruzzo e Molise, Teramo, Italy; 3 Directorate of Prevention, Food Safety, and Veterinary Public Health, Veneto Region, Venice, Italy; 4 Directorate of Prevention, Food Safety, and Veterinary Public Health, Emilia-Romagna Region, Bologna, Italy; 5 Istituto Zooprofilattico Sperimentale Lombardia ed Emilia-Romagna, Sezione di Reggio Emilia, Italy; 6 Bambino Gesù Children's Hospital, Rome, Italy; 7 Italian National Blood Centre, National Institute of Health, Rome, Italy; 8 Italian National Transplant Centre, National Institute of Health, Rome, Italy; 9 Italian Ministry of Health, Rome, Italy; 10 The members of the working group are listed at the end of the article

**Keywords:** West Nile Virus, One Health surveillance

## Abstract

In Italy, the 2018 West Nile virus transmission season started early with a high number of cases reported. One-Health surveillance, within the Italian West Nile national preparedness and response plan, detected viral circulation 9 days before symptom-onset of the first confirmed human case; triggering timely implementation of blood and transplant safety measures. This is an example of how functional coordination allows health authorities to use early warning triggers from surveillance systems to implement preventive measures.

In 2018, West Nile virus (WNV) transmission in endemic Provinces in Italy began early with higher human case counts of confirmed infection compared with previous years. Here the start of the transmission season is described together with the response triggered by the integrated surveillance activities.

## Early West Nile virus transmission in Italy 2018

**Figure 1:**
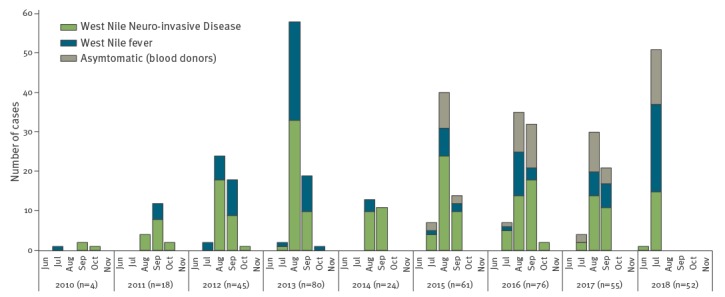
Confirmed human cases of West Nile virus infection notified in Italy, June 2010–July 2018 (n = 415)

**Figure 2:**
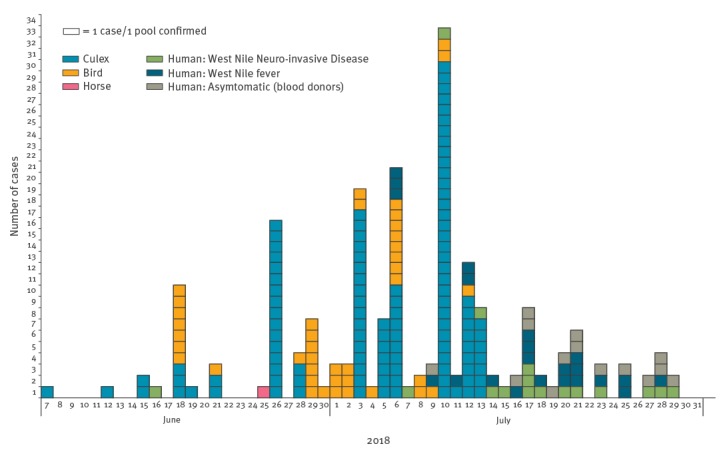
West Nile virus detection in *Culex* mosquitoes, birds, horses and humans, by date of detection or of symptom onset of West Nile fever, Italy, June–July 2018

In Italy, the earliest human cases of WNV infection are usually detected in July, with peaks in the number of cases in August–September (Figure 1). In 2018 however, the transmission season began earlier with the first detection of WNV in the Province of Rovigo (Veneto Region) [1] on 7 June from a pool of *Culex* mosquitoes; the first confirmed human case developed symptoms 9 days later in the same endemic Province (Figure 2).

111 confirmed positive mosquito pools, 40 confirmed infections in birds, one confirmed infection in a horse and 52 confirmed human infections.

**Figure 3:**
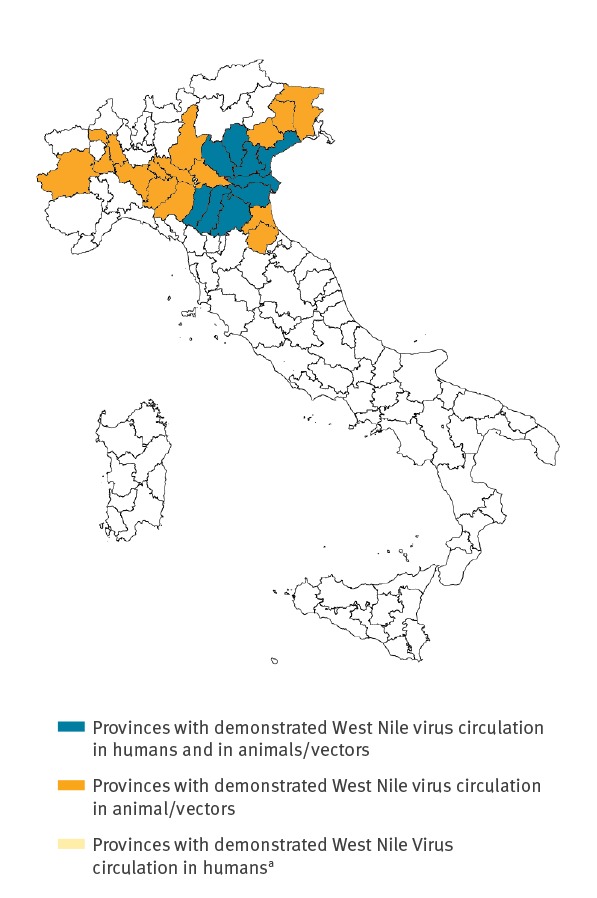
Provinces where West Nile virus infection was detected in mosquitoes/animals/humans, Italy, June–July 2018 (n = 25 provinces, of which 9 also reporting human infections)

^a^ No Provinces demonstrated West Nile virus circulation only in humans. To date human infections were reported only in Provinces where West Nile virus circulation in animals/vectors was also demonstrated.

As of 1 August 2018, 52 confirmed cases of human WNV infection were notified in the Veneto and Emilia-Romagna Regions. These include 16 cases of West Nile Neuro-invasive Disease (WNND), 14 cases of West Nile fever (WNF) and 22 asymptomatic infections. Two WNND cases died (case–fatality rate (CFR) for WNND: 12.5%). In all the affected Provinces, viral circulation was initially detected in mosquitoes and animals (equids and birds) and then later in humans, with no Provinces reporting only human cases (Figure 3). To date, only lineage 2 WNV has been identified in mosquitoes/animals. 

Since 2009, the National Blood Centre (CNS) [2] and the National Transplant Centre (CNT) [3] have introduced nucleic acid testing (NAT) to detect and so prevent transmission of WNV infection through donated substances of human origin (SoHO). In addition to NAT, the CNT has introduced IgG/IgM testing to screen donors of tissues, cells and organs. Province-level (NUTS3) triggers for SoHO safety measures are confirmed viral detection in mosquito pools, or confirmed infection in animals or humans.

To date, in 2018, all SoHO safety measures have been activated as a result of animal/entomological triggers. As of 1 August 2018, CNS and CNT issued 14 official notes, in some instances addressing several Provinces at the same time. Vector control measures, as specified in the National preparedness and response plan [4] and further described in derived Regional plans and guidelines [5-8], were implemented at municipal level in all the affected Provinces.

## West Nile virus epidemiology and integrated surveillance and response in Italy

Following the re-introduction of WNV in Italy in 2008 [9], it has caused cases of severe disease in humans every year. Only WNV lineage 1 was detected until 2011, when lineage 2 co-circulation was identified [10,11]. The disease is endemic in part of the country, mainly in Provinces located in a large valley in Northern Italy that hosts the basin of the largest Italian river (Po valley [12]). Sardinia, Sicily and the coastal Provinces of Lazio and Toscana are also endemic areas [4]. Transmission occurs mainly from bites of infected *Cule*x mosquitoes but can also occur through the transfusion/transplant of contaminated SoHO [13].

In Italy, WNV is managed through an annually revised plan [4] aiming to reduce the risk of transmission to humans by detecting viral circulation early and triggering both vector-control and SoHO safety measures. The plan, coordinated by the Italian Ministry of Health, defines data flow processes to facilitate a rapid response among relevant stakeholders including: the Istituto Zooprofilattico Sperimentale dell’Abruzzo e Molise (IZSAM), the National Institute of Health (ISS-Rome) that hosts CNS and CNT and the National Reference Laboratory for Arboviruses as well as the National surveillance of human cases of WNV, Regional Authorities, Regional Reference Laboratories for Arboviruses, the network of the Istituti Zooprofilattici Sperimentali and Local Health Units and Municipalities. Thanks to this plan, communication exchange between human-animal and entomological surveillance actors and other health authorities has been strengthened.

The plan also defines the National integrated human, animal and entomological surveillance (One Health Surveillance [14]) which is intensified at provincial level on the basis of seasonality and local epidemiology [4]. Since the first human outbreak of WNV in Italy, increased awareness of the disease has enhanced case detection. Combined with improved surveillance, this has led to increased reporting of WNF cases, along-side WNND cases and, since 2015, to the detection of asymptomatic WNV infections among blood donors.

## Conclusion

This year WNV transmission began earlier and with higher human case counts than in previous years in Italy. Compared with the same period 1971–2000, in June 2018 warmer temperatures and a higher cumulative rainfall were recorded [15]; possibly leading to more favourable conditions for mosquito survival and abundance and thus WNV transmission. At present, more human cases are expected until the end of the WNV transmission season. The situation is currently being monitored and preventive measures implemented as appropriate. Individual protection from mosquito bites remains the most important measure towards preventing WNV infection including the use of mosquito repellents, in accordance with the instructions indicated on the product label, wearing light coloured long-sleeved shirts and long trousers and sleeping or resting in screened or air-conditioned rooms [16].

In 2018, early WNV transmission has been also observed in other countries in South and South Eastern Europe [17]. In recent years changing patterns of transmission are being reported for several vector-borne diseases in Europe [18,19] and climate change has been implicated as a contributing factor [20]. In the face of these changes, the One Health surveillance system in Italy managed to maintain its early warning function and, as a consequence, to date during 2018, all SoHO safety measures were enforced before the first human case was reported in each affected Province. This surveillance system, whose economic return was demonstrated in Italian endemic areas [21], is providing early warning signals and those are being acted upon in a timely fashion by implementing preventive measures.

## Notes

**Working group:** The Working Group: C. Cenci, O. Bessi (Ministry of Health, Italy); V. Morabito (National Transplant Centre, Istituto Superiore di Sanità, ISS, Italy); G. Marano, I. Pati (National Blood Centre, Istituto Superiore di Sanità, ISS, Italy);C. Fortuna, M.E Remoli, R. Romi, F. Severini,, D. Boccolini, L. Toma, (Infectious Disease Department, Istituto Superiore di Sanità, ISS, Italy); M.C. Re (University of Bologna, Bologna, Italy); P. Angelini, A. Mattivi, C. Gualanduzzi (Regional Health Authority of Emilia Romagna, Bologna, Italy); F. Zanella, (Prevention and Public Health Unit, Directorate of Prevention, Food Safety, and Veterinary Public Health, Veneto Region, Venice, Italy); M. Claus (School of Specialization in Hygiene, Preventive Medicine and Public Health, University of Padua, Italy); P. Calistri, F. Iapaolo, M. Goffredo, G. Mancini (IZSAM); G. Capelli, F. Montarsi (IZSVE); M. Tamba (IZSLER); C. Casalone (IZSPLV).
